# A Framework for Inspiring COVID-19 Vaccine Confidence in African American and Latino Communities

**DOI:** 10.3390/vaccines10081319

**Published:** 2022-08-15

**Authors:** Zanthia Wiley, Lana Khalil, Kennedy Lewis, Matthew Lee, Maranda Leary, Valeria D. Cantos, Ighovwerha Ofotokun, Nadine Rouphael, Paulina A. Rebolledo

**Affiliations:** 1 Emory Department of Medicine, Division of Infectious Diseases, 100 Woodruff Circle, Atlanta, GA 30322, USA; 2 Hope Clinic of the Emory Vaccine Center, 500 Irvin Court #200, Decatur, GA 30030, USA; 3 Grady Healthcare System Infectious Disease Program, 341 Ponce de Leon Ave, NE, Atlanta, GA 30308, USA; 4 Hubert Department of Global Health, Rollins School of Public Health, 1518 Clifton Road, Atlanta, GA 30322, USA

**Keywords:** COVID-19 vaccines, COVID-19 vaccine hesitancy, African American, Black, Latinx, Latino, Hispanic, community-based organizations, migrant, immigrant, mistrust

## Abstract

The COVID-19 pandemic has disproportionately impacted racial and ethnic minority communities, particularly African American and Latino communities. The impacts of social determinants of health, structural racism, misinformation, and mistrust have contributed to a decreased COVID-19 vaccine uptake. Effective methods of addressing and combatting these barriers are essential. Accurate and targeted messaging delivered by trusted voices from community-based organizations, government health systems and organizations, and healthcare and academic systems is imperative. Outreach and communication should be culturally sensitive, provided in the preferred language of the community, flexible, and tailored for in-person and virtual outlets. This communication must also increase trust, combat misinformation, and inspire COVID-19 vaccine confidence. In this manuscript, we outline a framework for inspiring COVID-19 vaccine confidence in African American and Latino communities. These methods of targeted outreach should be considered and implemented for urgent and nonurgent community public health efforts beyond the COVID-19 pandemic (e.g., monkeypox) and as a framework to inspire vaccine confidence in those living in racial and ethnic minority communities globally.

## 1. Introduction

The COVID-19 pandemic has disproportionately impacted racial and ethnic minority communities, including non-Hispanic Black or African American (henceforward African American) and Hispanic or Latina/o/x (henceforward Latino) communities, resulting in marked COVID-related morbidity and mortality [1,2,3,4,5,6,7]. Racial and ethnic differences in COVID-19 vaccine uptake have also been reported. The United States (US) African American and Latinx populations had the lowest percentages of persons fully vaccinated against COVID-19 during the early months of vaccine availability [8]. A US adult survey noted a significantly higher COVID-19 vaccine uptake rejection rate among African American participants, and found that this association was mediated by medical mistrust [9]. Similar findings in another survey of US adults noted that Latino and African American respondents were more hesitant than US white individuals to receive a COVID-19 vaccine, more likely to state that they wanted to wait for more than a year, less likely to indicate that they wanted a vaccine immediately, and less likely to encourage their family to obtain the vaccine [10].

The reasons behind COVID-19-related health disparities among vulnerable communities are multilayered. Structural and systemic racism in the US stems from a history of enslavement of African American people, subjecting ethnic and racial minorities to inhumane medical and research practices; this planted the seeds of medical mistrust of healthcare systems and the government among many African American and Latino people [11,12]. African American persons are more likely to occupy essential worker roles (including vocational nurses, personal care aides, medical assistants, bus drivers, and flight attendants) [13]. Latino persons often live in multigenerational households, reside in densely populated urban communities, and are overrepresented in the lowest-paying essential industries, thus, increasing their risk of infection [14,15]. Latino and African migrant persons who have an undocumented immigration status often avoid seeking COVID-19 vaccines or care, due to fear of deportation when accessing those services [16,17].

**Figure 1:**
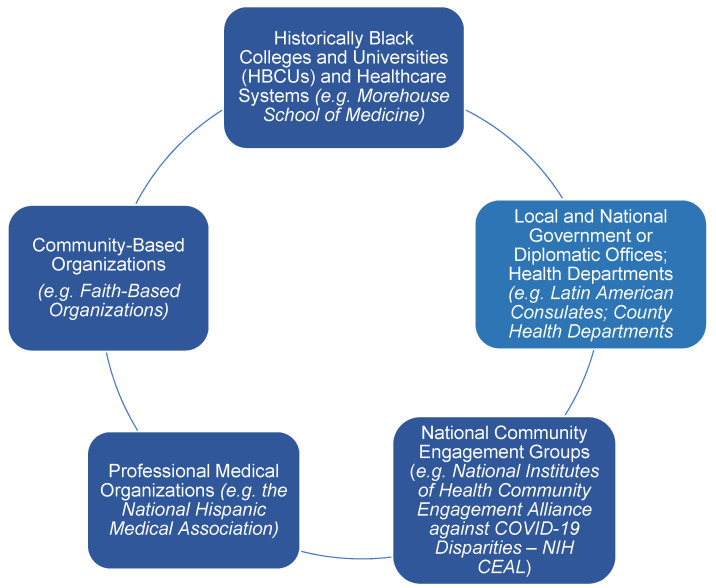
Key collaborators for inspiring COVID-19 vaccine confidence in African American and Latino communities.

There is an urgent need for culturally competent messaging and outreach efforts to prioritize African American and Latin communities in order to inspire COVID-19 vaccine confidence and enhance access to the vaccines. We describe a framework to connect community partners with government health systems and organizations, healthcare systems, and academic systems to mobilize efforts to address the disproportionate impact of COVID-19 on racial, ethnic, and socioeconomic minority groups (Figure 1). This framework describes key collaborators that serve as effective partners. Though the collaborations described are primarily US-based, these basic principles can be utilized for future public health illnesses affecting historically marginalized populations in other countries.

## 2. Where to Start: Start with Community-Based Organizations

To maximize the outreach to African American and Latino populations, it is essential to collaborate with established local community-based organizations (CBOs). Many CBOs are trusted voices with long-standing relationships with their communities. They also understand the social and cultural dynamics essential in building mutually beneficial opportunities for a COVID-19 vaccine outreach [18]. One example of an experienced community-based project is the Minnesota Immunization Networking Initiative (MINI) that provided free influenza vaccines to immigrant and racial/ethnic minority populations through community-based vaccination clinics [19]. The MINI stressed the importance of building relationships with community leaders, including involving them as full partners, having their clinics in community-based settings to bring the vaccines to the community, as well as reporting the outcomes to their community partners. Community-based health screenings of noncommunicable diseases that disproportionately impact African American and Latino communities (e.g., breast cancer) could also complement public health outreach education for COVID-19 [20].

## 3. Key Community Partnerships

*Local Community Advocacy Organizations*—These groups are generally trusted in the community, as they address the most pressing community needs and offer direct services. They also have an established audience, a large social network with other CBOs, and effective outreach methods. As an example “The Latino Community Fund Georgia” (LCF), a trusted local CBO in the Atlanta, Georgia metropolitan area, has built their community trust over time by organizing food banks, leading voter registration campaigns, helping families with census form completion, advocating for immigrant rights at the Georgia senate, and facilitating economic growth of Latino small businesses [21]. With the onset of the COVID-19 pandemic in 2020, the LCF formed a coalition with local health departments, Latino healthcare providers, and community volunteers to organize several COVID-19 “pop-up” testing and vaccination sites. These sites were developed to intentionally address the known barriers to healthcare that Latino people face. Specifically, these sites did not require preregistration (as some community members have difficulty navigating online forms), did not require a US-issued photo ID (as some individuals cannot obtain one due to their immigration status), and were in neighborhoods with a high Latino population density (to address transportation issues). To optimize outreach to the community, the LCF launched the “Unidos Georgia” [22]. Through photos and clear messaging, the campaign focused on the concept of a “community vaccinating community”. Available in English and Spanish, the “Unidos Georgia” website contains easy to understand COVID-19 information highlighting the benefits and safety of vaccines, a calendar of vaccination events, and a local map for COVID-19 resources. In addition, the LCF leveraged their social media presence to organize weekly webinars with Latino physicians to update the community about COVID-19.

*Faith-based organizations (FBOs)*—A key strategy to engage with African American and Latino groups is to partner with community agencies with deep ties with the faith community [23]. The Centers for Disease Control and Prevention (CDC), the Association of State and Territorial Health Official (ASTHO), and the Department of Health and Human Services surveyed 59 ASTHO member jurisdictions and four major US cities to evaluate territorial and state engagements with FBOs [24]. Of the 26 respondents, 92% (*n* = 24) reported that they engaged with FBOs to aid in the promotion of COVID-19 vaccination and, of the 24 respondents, 100% found that partnering with FBOs was valuable. The respondents worked with a diverse group of religious organizations, including churches, mosques, synagogues, and temples. In Atlanta, Georgia, the Fulton County Health Department partnered with five local African American churches, several Emory African American physicians, and Emory medical students. Together, they successfully vaccinated nearly 800 people on-site at their churches over several months. The LCF led similar COVID-19 vaccination campaigns in direct partnership with local Catholic churches located in geographical areas with high Latino population densities. The churches donated their space to conduct the vaccinations and were effective in spreading the word about the events to optimize attendance.

*Barber Shops and Hair Salons*—Barber shops and hair salons are staples in many African American communities, are visited frequently, and are a place for socialization and shared life experiences. Barber shops have been used to provide education on chronic illnesses, including obesity and cardiovascular diseases [25,26]. One randomized controlled trial enabling barbers to monitor blood pressures, become health educators, and promote follow-up with physicians resulted in improved hypertension control in participants [27]. A focus group of barber shop and hair salon owners in West Philadelphia, Pennsylvania, was conducted during summer 2020 to evaluate beliefs and attitudes around COVID-19 vaccines [28]. They found high COVID-19 vaccine hesitancy due to medical establishment mistrust, concern for accelerated vaccine development timelines, limited data on side effects, and racial injustice. They also found that recommendations from providers and safety profile transparency may aid in reducing COVID-19 hesitancy. In-person education, surveys, and leaving COVID-19 vaccine education materials at barber shops and salons are options to reach the community and promote COVID-19 vaccine confidence [25,26,28,29].

*Local Community Groups and Events*—A great way of connecting with a community is to join local community events and outreach programs to become a part of their COVID-19 vaccine outreach. For example, a yearly science festival for youth takes place in Atlanta, Georgia. During COVID-19, one of the virtual festival sessions provided was titled “COVID-19 Vaccines and Disparities in Black Communities: What You Need to Know” [30]. This offered attendees, particularly adults, the opportunity to learn about the COVID-19 illness, the inequities in the African American community, and the importance of vaccination. A Missouri community group, the “St. Louis Story Stitchers”, is comprised of young creative people of color (e.g., dancers, poets, and photographers) who use art to enact social change, while offering a foundation for community engagement and education [31]. In March 2022, they, along with the director of health for the City of St. Louis, presented a short performance and live-streamed discussion on COVID-19 and vaccines [32]. Neighborhood organizations also provide an effective way to disseminate COVID-19 vaccine information. Emory Latino physicians were invited to hold multiple small, in-person, and online conversations with members of Latino neighborhoods in the Atlanta area, where participants could voice their concerns and ask questions in a safe space.

*Local sports events*—Many athletes are respected members of their communities and may serve as trusted members to share COVID-19 vaccine information in African American and Latino communities. In October 2021, the North Carolina Department of Health and Human Services partnered with various organizations to promote COVID-19 vaccination among the Latino community during the Mexico versus Ecuador international soccer match that took place in Charlotte [33]. A fan meet-and-greet with a famed soccer player, media interviews, a mobile on-site COVID-19 vaccine unit, prize giveaways, informational tables from local Latino CBOs, and in-game education, including COVID-19 vaccine videos, digital graphics, and information about COVID-19 vaccines, was shared during game day. Bilingual staff and medical professionals were on site as well.

*Organizations Established with Migrant Communities*—When addressing COVID-19 vaccine hesitancy in migrant communities, it is important to note that issues beyond mistrust must be considered. For example, language barriers, cultural considerations, concerns for possible identification of undocumented status resulting in reporting to US Immigration and Customs Enforcement and resultant deportation, a lack of healthcare insurance, and lost wages due to the time needed to obtain a COVID-19 vaccine must all be considered during the process of inspiring vaccine confidence [34]. In March 2021, the Philadelphia Department of Health partnered with a local pharmacy and several immigrant service organizations to offer COVID-19 vaccines to immigrant residents. Providing COVID-19 vaccination in alternative trusted sites, including community centers, charities, shelters, and food banks, should also be considered [35].

*Employment Vaccination Events*—Many employers do not offer paid leave time for their employees to procure vaccinations. Other employment sites, such as farms or animal food-manufacturing factories, are in rural areas where access to traditional vaccination sites is limited or unavailable. Offering vaccinations to employees at their site of employment eliminates the need for transportation and missing work. In Georgia, several vaccination events led by the LCF and other CBOs have shown high community participation and acceptance of COVID-19 vaccination when conducted in tortilla factories, retail shopping centers, and on farms.

## 4. Other Key Partners

### 4.1. Local Health Departments

Local public health departments have played a crucial role in the COVID-19 vaccination uptake in African American and Latino groups. Many health departments provide COVID-19 vaccines ready to be administered to the population at no cost. Second, they provide CBOs with the expertise and training in setting up vaccination events, to guarantee patient and staff safety, the maintenance of vaccine cold-chain requirements and accountability, and assuring an adequate registration of vaccine administration in public records. For example, in Atlanta, a large sports stadium served as a massive-scale vaccination site. Attendance of Latino individuals remained low until local CBOs advised the Fulton County Board of Health to place a sign outside of the stadium indicating that all people were welcome, regardless of immigration status. After this, the attendance of Latino individuals increased substantially. As stated above, the Fulton County Board of Health also partnered with African American churches in Atlanta, which resulted in successful COVID-19 vaccine drives. In November 2021, the City of St. Louis Department of Health held a virtual town hall entitled “COVID-19 Vaccine: The Truth about the Pediatric Vaccine” to allow parents and guardians the opportunity to have their questions answered [36]. They used their town halls as a bidirectional forum to allow their community to receive firsthand information from trusted healthcare advisors, as well as to hear from community residents about their COVID-19 vaccine perceptions, knowledge, motivations, and beliefs in addition to potential and actual barriers to receiving the vaccine.

### 4.2. Local and National Governments or Diplomatic Offices

Community members have long engaged with their local government officials via town halls, congressional briefings, and community events. Local government officials and their offices are also potential venues of reaching the community to inspire confidence in COVID-19 vaccines.

*Latin American Consulates in the United States*—Members of the Latino community have struggled to access reliable and consistent healthcare services in the US, even before the onset of the COVID-19 pandemic. For years, initiatives stemming from the Consulates of Mexico and other Latin American nations have provided health preventive services to meet the needs of undocumented and Spanish-speaking communities in the US. Examples include the “Ventanilla de Salud” (VDS), a pilot project funded by the United States–Mexico Border Health Commission and the Health Initiative of the Americas from the University of California, aimed at narrowing health disparities for Mexican migrants seeking assistance from the Mexican Consulate in San Diego and Los Angeles, California [37]. Since its inception in 2003, the VDS has expanded to 24 states and has been instrumental in providing access to COVID-19 testing and vaccinations for the Latino community. By providing linguistic and culturally appropriate educational materials and care, and prioritizing undocumented individuals and mixed-status families, initiatives such as VDS should serve as models for how to create safe and trusted spaces for vulnerable communities. This initiative also provides a safe space for undocumented immigrants to receive COVID-19-related care without the fear of deportation.

### 4.3. National Community Engagement Groups

*The National Institutes of Health (NIH) Community Engagement Alliance (CEAL) against COVID-19 Disparities*—NIH CEAL—works closely with the communities hit hardest by COVID-19 [38]. Their website contains educational materials in English and Spanish, and the materials are friendly to the general population, with available resources including reports/guides, toolkits, facts sheets, infographics, videos, and websites [39]. For community members who engage with their communities via social media, there are also predeveloped social media messages that can be copied and used via social media messages to encourage the spread of accurate information about COVID-19 vaccines.

### 4.4. Professional Medical Organizations

*The National Medical Association (NMA)*—The NMA, founded in 1895, is the oldest and largest US national organization representing African American physicians [40,41]. Their services include advocacy and community health education targeting African American and medically underserved populations. The NMA COVID-19 task force supported the US FDA approval for emergency use authorization for the SARS-CoV-2 vaccines and have been vocal on the importance of equitable distribution of culturally sensitive information [42]. The NMA has over 100 state and local affiliate societies within six geographic regions, allowing for program implementation ranging from a national to a local impact. For example, the Atlanta Medical Association (a local NMA society) hosted a series of virtual sessions available to the public titled the “Ask a Black Doctor” series, which included informational sessions on COVID-19 vaccines where community members interacted directly with African American physicians to obtain answers to their questions [43].

*The National Hispanic Medical Association (NHMA)*—The NHMA, established in 1994, represents the interests of 50,000 Hispanic physicians in the US, and is dedicated to improving the health of Hispanic persons and eliminating health disparities [44]. The NHMA has offered multiple COVID-19 vaccine educational opportunities, including a Nebraska chapter COVID-19 vaccine soccer clinic and COVID-19 virtual briefing sessions (e.g., session titled “COVID-19 Isn’t Over Yet: Increasing Vaccinations Among Latinos in Overlooked Hotspots”). The NHMA also offers the opportunity to become a champion in the “Vaccinate for All/Vacunas Para Todos” campaign, which allows both healthcare professionals and community leaders to share trustworthy resources with Latinx community members [45]. These resources include “Boots-On-The-Ground” advocacy opportunities, where bilingual physicians answer questions on COVID-19 vaccination, and champions can seek out media training and have their media efforts amplified by the campaign’s platform.

### 4.5. Historically Black Colleges, Universities, and Academic Health Systems

*Historically Black Colleges and Universities (HBCUs)*—For over one hundred years, HBCUs have served their communities (and many HBCUs are located directly within marginalized and red-lined communities). Much of the service to the community is provided by members of historically African American fraternities and sororities. The National Pan-Hellenic Council, commonly referred to as the “Divine Nine”, includes members of historically African American sororities and fraternities with a legacy of promoting equity, social justice, and service [46]. The CDC Foundation connected with Philadelphia sororities and collaborated on a social media video encouraging COVID-19 vaccination and mask wearing [47]. They also developed a webinar series titled “COVID Conversations” featuring local African American physicians; the first webinar focused on COVID-19 vaccine safety, effectiveness, and equity. One sorority chapter developed a COVID-19 response team and partnered with the city of Philadelphia, the Black Doctors COVID-19 Consortium, and the Black Clergy. Their volunteers secured appointments for COVID-19 vaccines for nearly 400 people in the Philadelphia area. As part of HBCU week in September 2021, another sorority at Howard University and Morgan State Universities hosted free COVID-19 vaccinations and tests on campus and at a local church [48].

*Historically Black Healthcare Systems*—Advocacy work extends beyond HBCUs to historically African American medical schools as well. The Morehouse School of Medicine utilized multiple strategies to ensure that high-risk, hard-to-reach populations received COVID-19 vaccines via a mobile vaccination unit that was able to serve those who reside in rural communities and essential workers [49]. They also hosted a YMCA back-to-school COVID-19 vaccine event, as well as a Latino community fund COVID-19 vaccine event. The Meharry Medical College COVID-19 mobile vaccine program (MMC-MVP) comprised of infectious disease specialists, nurse practitioners, and community engagement staff, providing COVID-19 vaccines to Middle Tennessee communities, which allowed persons who were homebound, had a lack of access to public transportation, and those with decreased access to medical providers or vaccine clinics the opportunity to get vaccinated [50]. The MMC-MVP partnered with Vanderbilt University School of Nursing COVID-19 vaccine strike teams as well other CBOs and FBOs. The MMC-MVP provided almost 5000 COVID-19 vaccines to targeted, marginalized communities during the September 2021 COVID-19 Tennessee surge.

Engagement within African American and Latino communities is not limited to HBCUs and predominantly minority-serving institutions. Many academic medical centers have established relationships (e.g., via community–academic partnerships), with surrounding communities. These community partnerships are excellent starting points for beginning communication and collaborations with the Latino and African American communities [51].

## 5. Discussion

The COVID-19 pandemic exposed and accentuated preexisting inequalities experienced by Latino and African American populations. Despite suffering a disproportionate burden of COVID-19-related morbidity and mortality, as well as an inequitable impact in the areas of education, mental well-being, and financial security, these communities had the lowest level of vaccine acceptance during the initial months following the availability of COVID-19 vaccines [52,53]. Community involvement should be part of the backbone for promoting health equity and empowering ethnic and racial minorities to form partnerships with institutions and organizations committed to systemic change to advance the rights and health of communities of color.

Among African American and Latino groups, a common barrier to the COVID-19 vaccine uptake was the belief that vaccines were developed too quickly, raising concerns about their safety and legitimacy. Intentionally increasing the representation of African American and Latino participants in COVID-19 vaccine clinical trials may serve as an additional tool to overcome this and other misconceptions about COVID-19 vaccines, while, simultaneously, familiarizing them with research. An increased representation of African American and Latino professionals in clinical care and research is also key, as they can serve as advocates for the communities they represent. At research sites, intentionally hiring staff who are part of the most affected racial/ethnic group facilitates communication with potential and enrolled participants, not only due to language barriers, but also an implicit understanding of the participants’ priorities and struggles. Of utmost importance is the collaboration of all entities with CBOs and their community workers who have served with steadfast commitment (even prior to the COVID-19 pandemic) is crucial.

In this manuscript, we summarized some of the collaborative, multidisciplinary collaborations that resulted in an increased COVID-19 vaccine uptake by African American and Latino groups in the US. Although specific needs may have been different between these two groups, there were several common strategies that yielded good results (Table 1). These included: 1. begin the process with listening to the affected communities and their trusted local CBOs to learn about their priorities and preferences (language, flexible timing for vaccinations, and location of vaccination sites), as well as main uptake barriers; 2. use this information as direct guidance to plan and implement education and vaccination campaigns; 3. use clear, easy-to-understand language in education and vaccination outreach campaigns; 4. avoid risk- or fear-focused messages and focus on benefits and safety of vaccines; 5. leverage the CBOs’ established personal, organizational, and online social networks to disseminate information; 6. use physical or online venues identified as safe spaces by the community for vaccination sites, to overcome fear and optimize open conversations; and 7. form a coalition of CBOs, health departments, academic institutions, and other local government organizations who are committed to increasing COVID-19 vaccination in their communities and leverage each group’s area of expertise. Methods to increase COVID-19 vaccination confidence in African American and Latino communities must include analyses of beliefs, norms, misinformation, and preconceived notions, and strategies must incorporate the removal of vaccine access barriers, customizing education to the community, and intimate engagement with the community [54]. In this manuscript, we offered a framework for inspiring COVID-19 vaccine confidence in African American and Latino communities. This framework can be used to inspire confidence in COVID-19 vaccine boosters, inspire vaccine confidence in other public health emergencies (e.g., monkeypox), and inspire vaccine confidence in minoritized communities in other countries. At the center of inspiring vaccine confidence, in any country and with any vaccine-preventable disease, is leveraging established community leaders and organizations and, most importantly, establishing trust.

## Figures and Tables

**Table 1:** Strategies to promote COVID-19 vaccine uptake in African American and Latino communities.

Listen to effected communities and their trusted local community-based organizations (CBOs). Use this information as direct guidance to plan and implement education and vaccination campaigns. Use clear, easy-to-understand language in education and vaccine outreach campaigns. Avoid risk- or fear-focused messages and focus on the benefits and safety of vaccines. Leverage the CBOs’ established personal, organization, and online social networks to disseminated information. Use physical or online venues identified as safe spaces by the community for vaccination sites (to overcome fear and optimize open conversations). Form a coalition of CBOs, health departments, academic institutions, or other local government organizations who are committed to increasing COVID-19 vaccination in their communities and leverage each group’s area of expertise.

## Data Availability

Not applicable.
